# Modulation of early life gut microbiota through inclusion of earthworms and vermicompost in broiler diets

**DOI:** 10.1016/j.psj.2025.105121

**Published:** 2025-04-01

**Authors:** Muhammad Zeeshan Akram, Oyekunle John Oladosu, Nadia Everaert, Cornelia C. Metges, Gürbüz Daş

**Affiliations:** a Nutrition and Animal-Microbiota Ecosystems Laboratory, Department of Biosystems, KU Leuven 3000, Heverlee, Belgium; b Research Institute for Farm Animal Biology (FBN), Dummerstorf 18196, Germany

**Keywords:** Challenge diet, Early life, Functional profiling, Microbial diversity

## Abstract

We previously showed that providing earthworms (**EW**) to broilers during early life can mitigate dietary challenges induced by soluble non-starch polysaccharides (**NSP**). However, whether the positive effects of providing EW are associated with changes in the gut microbial communities of broilers was not studied. This follow-up study investigated the influence of providing EW and vermicompost (**VC**) on gut microbiota diversity in broilers fed either a standard corn-soy based diet as positive control (**CON+**) or a diet rich in NSP as negative control (**CON-**). A total of 120 newly hatched male birds of Cobb-500 genotype were examined in two periods (**P**), each lasting 8 days. In P1, birds were divided into four groups: two groups received the CON+ diet (n = 30 each), the third group received CON+ plus 1 % EW (**CON+EW**, n = 30), and the last group received CON+ supplemented with 1 % VC (**CON+VC**, n = 30). Half of the birds in each group were euthanized at the end of P1, and ileal digesta were collected for microbiota analysis. In P2, one of the CON+ groups from P1 continued the same diet, while the remaining groups were switched to dietary challenge either NSP supplemented negative control CON- (n = 15), or **CON-EW** (n = 15) or **CON-VC** (n = 15). At the end of P2, the remaining birds in all groups were euthanized for ileal digesta collection. Microbial composition was assessed using 16S rRNA gene sequencing. In P1, the CON+VC exhibited a significantly higher Chao1 index compared to the CON+ and CON+EW (*P* < 0.05). In P2, α-diversity metrics remained unchanged across groups (*P* < 0.05), although the Chao1 index in the CON+ showed a trend toward an increase (*P* = 0.078). Analysis of β-diversity highlighted significant differences between dietary groups in P2 (*P* = 0.001). Further analysis identified differentially enriched genera, revealing that *Enterococcus* was prominent in the CON+ during P1, while *Lactobacillus* was significantly higher in the CON+EW group. In P2, the CON-EW group exhibited increased *Lactobacillus* abundance while *Escherichia-Shigella* was overrepresented in the CON- group. Functional analysis showed that the CON-EW and CON-VC diets enriched the pathways related to Nicotinamide Adenine Dinucleotide biosynthesis and fermentation to acetate and lactate, whereas the CON- increased the biosynthesis of enterobactin and aerobactin. In conclusion, dietary earthworm supplementation positively influenced gut microbiota composition and predicted functions in response to dietary challenges.

## Introduction

Early access to beneficial microorganisms is particularly critical for broilers which rely almost solely on the innate immune responses when newly hatched (Rubio 2019). A diverse and well-established gut microbiome can strengthen the immune system, support efficient digestion, and protect against pathogenic bacteria (Ducatelle et al. 2023). In intensive production systems, where chickens are raised in controlled environments with high hygiene standards, they often lack access to a diverse microbiome. Although this reduced exposure to pathogens is potentially expected to have preventive benefits, it may result in limited microbial phylogenetic diversity compared to chickens raised in extensive systems or environments with less strict hygiene practices (Marcolla et al. 2023; Zenner et al. 2021).

Broilers in extensive production systems are exposed to a variety of microorganisms from soil, insects, and decomposing organic matter, which facilitates more diverse gut colonization early in life (Marcolla et al. 2023). Considering the evidence supporting early and optimal microbial colonization, the relatively reduced microbial diversity in intensively farmed broilers may therefore contribute to digestive challenges and slower microbial adaptation to dietary changes. Broilers have genetically been intensively selected for fast growth and higher feed efficiency, which may however limit their ability to overcome environmental and immunological challenges (Rauw et al. 1998; Zuidhof et al. 2014). The enhancement of broilers’ microbiome, particularly in early life is for these reasons pertinent.

The addition of earthworms (EW) to broiler diets provides an opportunity to increase or stimulate microbial diversity in modern commercial poultry farms, as EW have considerable microbial activity in their gut (Curry and Schmidt 2007). Earthworms are a rich source of protein (Lawrence and Millar 1945) with balanced amino acids, and essential minerals (Kavle et al. 2023). Earthworms inherently harbour a vast number of microorganisms (Curry and Schmidt 2007; Edwards and Fletcher 1988) characterized by high diversity (Aira et al., 2022). Similarly, vermicompost (VC), the excreta of EW (Edwards and Fletcher 1988), contains high levels of humic substances and microbial biomass (Domínguez et al. 2019), which when fed to chickens can suppress growth of pathogenic bacteria (Spencer et al. 1998). With the growing emphasis on production systems aligned with circular bioeconomy principles, bio-based feed materials, including EW and VC, hold the potential to enhance gut microbial colonization in modern broilers, mimicking natural microbial exposure.

Non-starch polysaccharides (NSPs) in feed, particularly when grains such as wheat, barley, and rye are used can pose nutritional challenges (Bach Knudsen 2001). While NSPs can provide energy to the host through microbial fermentation, chickens lack the endogenous enzymes needed to break down these complex carbohydrates. As a result, NSPs increase intestinal viscosity, disrupt nutrient absorption, and impair growth performance. Increased gut viscosity slows the passage of digesta, traps nutrients, and limits enzyme access, leading to reduced feed efficiency (Bedford 2000). Bio-based strategies that modulate gut microbiota via dietary supplementation are gaining interest. Earthworm and VC, with their rich microbial content, may offer a bio-based solution for early gut colonization and mitigating dietary stress and inefficiency associated with digestion of fibre. By promoting the early establishment of a diverse and functional microbial community, EW and VC supplementation could enhance the fermentation of NSPs and reduce the negative impact of these fibres on broiler performance. Our recent findings demonstrated that feeding broilers with EW was associated with a reduction in pasty vent and increased caecal size, suggesting improved gut fermentation capacity and microbial health (Daş et al. 2024). Vermicompost on the other hand, improved bird growth when fed with a standard diet, whereas it intensified anti-nutritional effects when included in NSP-supplemented diets. These results suggest that EW supplementation may enhance microbial resilience in the gut, facilitating chickens’ adaptation to the challenges posed by NSP-enriched diets. It remained, however, unclear if the positive effects of EW supplementation were directly associated with the changes in gut microbial communities in broilers. This follow-up study aims to evaluate the impact of dietary EW and VC supplementation on the ileal microbial communities in broilers fed standard corn-soybean-based diets without or with additional NSP over two periods, each lasting 8 days (**d**).

## Materials and methods

### Experimental design

A subset (n = 120) of male Cobb-500 chicks (Avimex GmbH, Brüterei Wiesenena, Wiedemar, Germany) from a previously described larger-scale study (Daş et al. 2024) was used in this experiment. The larger-scale foundational experiment with 480 birds was carried out in two periods (P), each lasting 8 d, and was replicated twice with two different batches of birds. A simplified experimental design used for this follow-up study is given in Supplementary Figure 1, while ingredients and chemical composition of experimental diets are summarized in Supplementary Table 1.

In P1, the newly hatched birds were assigned to 4 groups, each represented with 6 pen replicates (n=30 birds per group). The birds in two groups were fed a corn-soybean based control diet (**CON+**) in mash form without EW or VC supplementation for 8 d (i.e. P1). The third group also received the CON+ diet with an additional supplementation of fresh EW on top (i.e. **CON+EW**). The EW supplementation corresponded to 1 % of daily dry matter (**DM**) intake of CON+EW birds from the previous day. Fresh EWs (*Dendrobaena veneta*, i.e. European Nightcrawler) were offered to the birds on a feeding plate in the mornings at the same time. The fourth group received the CON+ diet supplemented with VC at 1 % in DM (i.e. **CON+VC**). At the end of P1, half of the birds in each group (15 birds per group) were euthanized, and ileal digesta samples were collected for microbiota analysis as described below. Both EW and VC used in this study were sourced from Martin Langhoff SUPERWURM e.K., Düren, Germany.

From d9 to d16 (i.e. P2) the remaining birds on one of the CON+ groups from P1 stayed on the same CON+ diet (CON+, n =15), whereas the second CON+ group was switched to a high NSP supplemented negative control diet (**CON-**). For production of the CON- diet with a higher amount of NSP, approximately 50% of corn provided in the CON+ was replaced with wheat, barley and rye in equal proportions (Supplementary Table 1). The birds receiving EW or VC on top of CON+ diet in P1 (i.e. CON+EW and CON+VC) were switched to CON- diet with their corresponding EW or VC supplementations in P2 (i.e. **CON-EW** and **CON-VC**, respectively). In the end of P2 (d16) all the birds in four groups were stunned and euthanized for ileal digesta sampling and the experiment was terminated. Further details of the experiment, methods used for chemical analysis of the diets described earlier (Daş et al., 2024).

The experiment was carried out in compliance with European Union regulations regarding animal welfare, including guidelines for animal care, handling, stunning, and slaughter. All sampling procedures were conducted by personnel who received appropriate training. Ethical clearance for the study was secured from the State Ethics Committee for Animal Experiments (Mecklenburg-Western Pomerania State Office for Agriculture, Food Safety and Fisheries, Germany; approval number: 7221.3-2-015/19-1).

### Microbial DNA extraction and V3-V4 16S rRNA gene amplicon sequencing

Digesta from distal ileum was collected from 15 birds per group at slaughter (i.e. d8 and d16) and stored at -80°C for subsequent microbial analysis. DNA was extracted from ileal digesta samples using the QIAamp PowerFecal Pro DNA Kit (Qiagen Benelux B.V., Venlo, the Netherlands), following the manufacturer's standard protocol. The concentration and quality of the extracted DNA were assessed using a Nanodrop 2000 spectrophotometer (Thermo Fisher Scientific, Waltham, MA) for quantification and 1% agarose gel electrophoresis for quality control. Out of the initial 15 samples per group, DNA from the following samples met the quality and concentration thresholds for sequencing: 9 samples from the CON+ group, 12 from the CON+EW group, and 13 from the CON+VC group in P1; and 10 from the CON+ group, 9 from the CON- group, 13 from the CON-EW group, and 10 from the CON-VC group in P2.

Sequencing libraries were prepared to target the V3-V4 regions of the 16S rRNA gene, using primers 341F (5′-CCTAYGGGRBGCASCAG-3′) and 806R (5′-GGACTACNNGGGTATCTAAT-3′), with sample-specific barcode sequences. The resulting libraries were sequenced on an Illumina NovaSeq 6000 platform, which generated 250 bp paired-end raw reads. To ensure quality control during sequencing, DNA from water was included as a negative control.

### Data processing, quality assessment, bioinformatics and statistical analysis

Raw sequences were processed using Qiime2 (v2024.2), where quality filtering, trimming, and demultiplexing were performed with default parameters. Following pre-processing, reads were processed using DADA2 to remove low-quality sequences (with Phred score < 20).The first 11 bases were trimmed, and reads were truncated at 398 bp to improve the overall quality. An amplicon sequence variant (ASV) table was then generated. Taxonomic classification of the ASVs was carried out using the Naïve Bayes Classifier method against the SILVA database (release 138) with a 99 % shared identity threshold. For statistical analysis, Qiime2 artifacts were imported into R (v4.2.3, R Foundation, Vienna, Austria) to make phyloseq object (v1.40.0). Downstream analysis focused on bacterial domain sequences, and the negative control was excluded from the dataset. In the dataset, ASVs contributing less than 0.01 % of the total relative abundance across all samples were filtered out. Ileal amplicon sequencing generated 7,026,819 reads, with 91,257 ± 8593 (mean ± SD) reads per sample. After quality filtering, 6,324,137 reads remained, with an average of 82,132 ± 7734 reads per sample (Range: minimum = 57,940 and maximum = 100,709). A total of 1870 ASVs were identified in both periods.

Alpha and Beta diversities were calculated using the phyloseq package (v1.40.0) in R. Alpha diversity metrics, including Chao1, Shannon, and Simpson indices, were calculated after rarefying the ASV table to a minimum sample depth of 57,940 sequences to assess microbial richness, overall diversity, and evenness. Beta diversity was calculated using the Bray-Curtis dissimilarity matrix, and the results were visualized through principal coordinate analysis (PCoA). For the sake of simplicity, sampled obtained from the birds on two identical CON+ diets in P1 were pooled and considered as a single group. Differences in beta diversity between groups within each period were evaluated using a non-parametric permutational multivariate analysis of variance (PERMANOVA) with 9999 permutations using adonis2 function from the vegan package (v2.6.4) in R. Differential abundance of microbiota at the genus level between groups was determined using Linear Discriminant Analysis (LDA) Effect Size (LEfSe) in R using the microbiomeMarker package (v1.18.0). Default parameters of LDA ≥ 2.0 and a significance threshold of P < 0.05 were used. The obtained *P* values were further adjusted for false discovery rate (FDR) using the Benjamini-Hochberg method, and the results were visualized based on Log10 (LDA score). The non-parametric Kruskal-Wallis test was applied to compare differences in alpha-diversity, relative abundance, and predicted functional profiling across multiple variables, while the Wilcoxon rank sum test was used for pairwise comparisons among dietary treatments within a period. An additional comparison using Wilcoxon test was performed only for CON+ diet between P1 and P2 to separate time/age related effects of birds from the dietary effects of CON+.

Functional profiling of microbial communities was predicted using PICRUSt2 (Phylogenetic Investigation of Communities by Reconstruction of Unobserved States) based on the 16S rRNA gene sequences. Functional predictions were built on the MetaCyc Metabolic Pathway Database (https://metacyc.org/). Heatmaps were generated in R using the pheatmap package (v1.0.12) to visualize sample variability in predicted metabolic pathways. Values for predicted metabolic pathways were row-scaled, and a two-way hierarchical clustering was performed using Pearson's correlation distance and the Ward clustering method. To gain further insight into the functions of abundant bacterial genera, Pearson's correlation analysis was performed to assess the relationships between differentially abundant bacterial genera and significantly altered predicted metabolic pathways, using data pooled across three groups in P1 and four groups in P2.

## Results

### Alpha and beta diversity

**Fig. 1:**
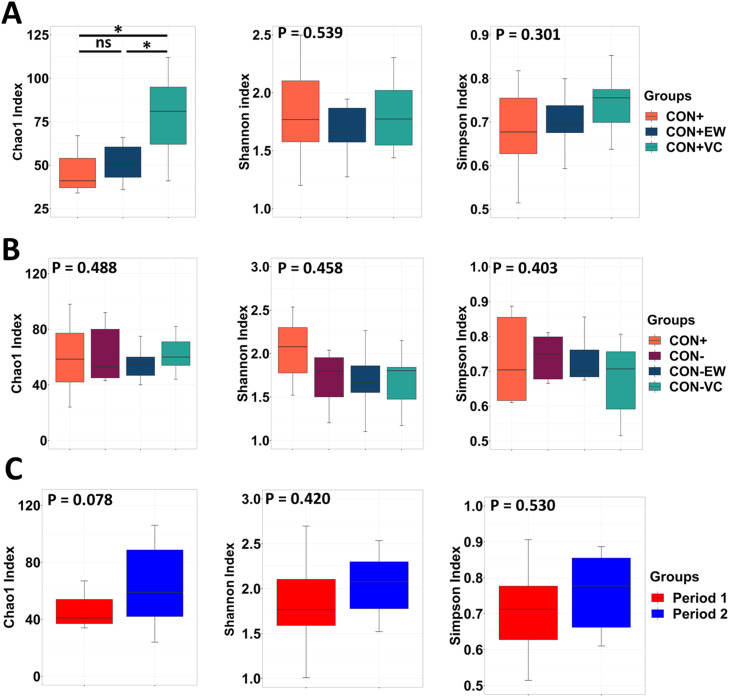
Alpha diversity metrics (Chao1, Shannon, and Simpson indices) of the ileum microbiota across different groups during Period 1 (A) and Period 2 (B), and within the CON+ group between Period 1 and Period 2 (C). Statistical significance was assessed using the non-parametric Kruskal-Wallis test for comparisons among more than two groups and the non-parametric Wilcoxon test for pairwise comparisons.

**Fig. 2:**
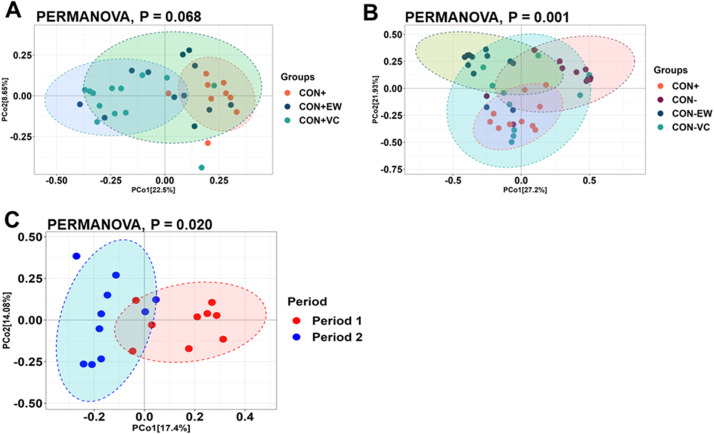
Principal coordinate analysis (PCoA) of beta diversity based on the Bray-Curtis dissimilarity matrix of ileum microbiota in broilers in (A) Period 1 and (B) Period 2, or in broilers fed CON+ diet in both Period 1 and Period 2. Multivariate effects of treatments on beta diversity were evaluated using non-parametric permutational multivariate analysis of variance (PERMANOVA).

In P1, the CON+VC diet induced a significantly higher Chao1 index compared to both the CON+ and CON+EW diets (Fig. 1A, *P* < 0.05). Correspondingly, Bray-Curtis distance-based beta diversity analysis revealed a trend toward group dissimilarities in P1 (Fig. 2A, PERMANOVA, *P* = 0.068). In P2, alpha diversity metrics were not significantly affected by the treatments and remained consistent across groups (Fig. 1B, *P* > 0.05). However, beta diversity was significantly affected by the dietary treatments in P2, resulting in clear differences between groups (Fig. 2B, PERMANOVA, *P* = 0.001). When evaluating the age/time-dependent effects within the CON+ group between the two periods, the Chao1 index showed a tendency to increase in P2 compared to P1 (Fig. 1C, *P* = 0.078). In line with this change, a significant (*P* = 0.020) time-dependent beta diversity difference in microbiota composition of CON+ fed birds was observed between the two periods (Fig. 2C).

### Core microbiota composition

Overall, compositional analysis consistently identified Firmicutes as the predominant (>96 %) phylum in ileal samples of the birds across all groups during both periods (Table 1). In P1, Firmicutes accounted for approximately 99 % of the total relative abundance, while in P2, their presence ranged from 96 % to 98 % (Table 1). Following Firmicutes, the next dominant phyla across both periods were Proteobacteria, Actinobacteriota, Cyanobacteria and Bacteroidota. The relative abundance of Actinobacteria was significantly lower in the CON+ group compared to the CON+EW and CON+VC groups in P1 (FDR < 0.003), while Cyanobacteria were significantly lower in the CON-EW group compared to all other groups in P2 (FDR = 0.048).

**Table 1:** Relative abundance of bacterial phyla in the ileum of experimental broiler groups in two periods.

Period 1 (d1-8)	CON+	CON+	CON+EW	CON+VC	FDR value
Firmicutes	99.71 ± 0.501	-	99.71 ± 0.449	99.55± 0.361	0.108
Proteobacteria	0.18 ± 0.369	-	0.06 ± 0.088	0.08 ± 0.069	0.534
Actinobacteriota	0.01 ± 0.0181^b^	-	0.08 ± 0.084^a^	0.19 ± 0.151^a^	0.003
Cyanobacteria	0.11 ± 0.255	-	0.14 ± 0.277	0.11 ± 0.218	0.786

Period 2 (d9-16)	CON+	CON-	CON-EW	CON-VC	
Firmicutes	96.64 ± 6.067	97.99 ± 2.821	97.80 ± 5.081	98.20 ± 1.647	0.335
Proteobacteria	2.28 ± 5.604	1.34 ± 2.584	1.39 ± 4.721	1.19 ± 1.571	0.331
Actinobacteriota	0.81 ± 2.566	0.22 ± 0.409	0.75 ± 2.289	0.293 ± 0.466	0.502
Cyanobacteria	0.22 ± 0.546^a^	0.30 ± 0.319^a^	0.05 ± 0.068^b^	0.255 ± 0.560^a^	0.048
Bacteroidota	0.03 ± 0.094	0.15 ± 0.265	0.01 ± 0.013	0.02 ± 0.047	0.253

Total number of birds sampled for ileal digesta sampling in this study was N = 120 (i.e. 15 birds / group in each period). Number of samples used for statistical analyses after DNA quality control was as following: Period 1: CON+ (n=9), CON+EW (n=12), CON+VC (n=13); Period 2: CON+ (n=10), CON- (n=9), CON-EW (n=13), and CON-VC (n=10).

**Abbreviations**: **CON+**: positive control diet; **CON-**: negative control diet; **CON+VC**: positive control diet supplemented with 1 % vermicompost; **CON-VC**: negative control diet supplemented with 1 % vermicompost in dry matter. Data were analyzed with Kruskal-Wallis test and pairwise comparison was performed with Wilcoxon test (P< 0.05). The obtained p values were adjusted for false discovery rate using Benjamini-Hochberg method.

Data are presented as means ± SD.

**Fig. 3:**
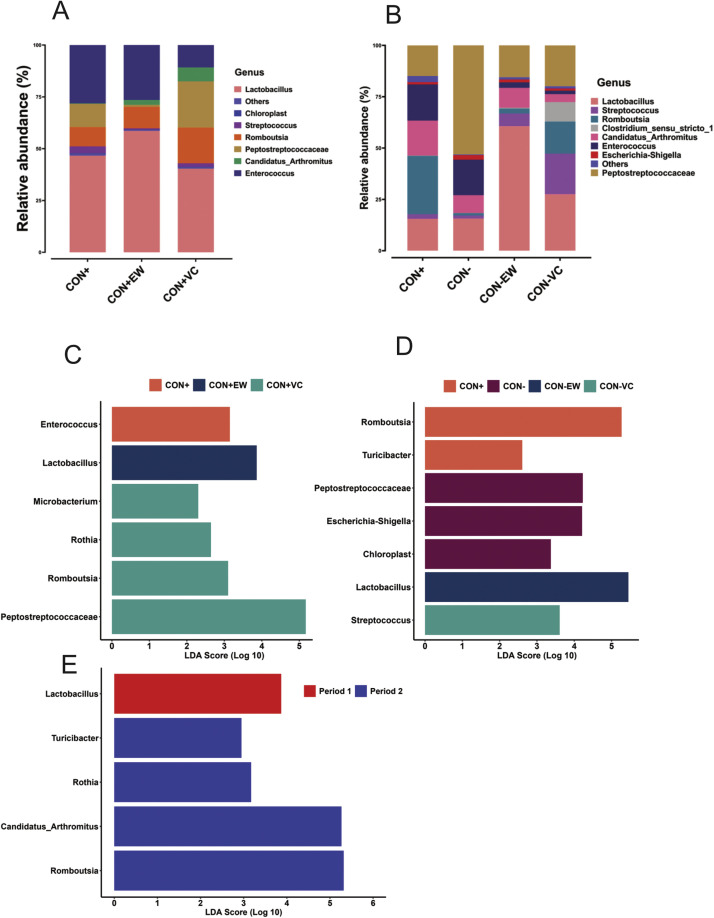
Relative abundance of bacterial genera in ileal microbiota: (A) in broilers fed different diets in period 1 and (B) in period 2. LEfSe results showing differentially abundant bacterial genera: (C) in broilers fed different diets in period 1 (D) and in period 2, and (E) in broilers fed CON+ diet in both Period 1 and Period 2. Only genera with an FDR ≤ 0.05 and an absolute LDA value > 2 are presented.

At the genus level, ileal microbiota displayed a distinct predominance of *Lactobacillus* in P1, comprising 40 % to 59 % of the total relative abundance, followed by *Enterococcus* (9 % to 17 %) and unclassified *Peptostreptococcaceae* (1 % to 22 %, Fig. 3A). In P2, the dominant genera included *Lactobacillus* (15 % to 60 %) and unclassified *Peptostreptococcaceae* (14 % to 53 %), along with *Romboutsia* and *Streptococcus*, which ranged from 1 % to 28 % and 2 % to 20 %, respectively (Fig. 3B). It is of note that in birds fed CON+EW in P1 and CON-EW in P2 *Lactobacillus* was the most dominant among all genera analysed.

### Differential abundance of bacterial genera

LEfSe analysis was used to identify specific bacterial genera that were differentially enriched among the groups within each period. Using a threshold of LDA of 2.0 and a FDR cut-off value of 0.05, a total of six bacterial genera were identified as differentially enriched across the three groups in P1 (Fig. 3C). Among these, *Enterococcus* was significantly enriched in the CON+ group, while *Lactobacillus* demonstrated higher abundance in the CON+EW group. Additionally, *Microbacterium, Rothia, Romboutsia,* and unclassified *Peptostreptococcaceae* were found to be enriched in the CON+VC group.

In P2, the LEfSe analysis identified seven bacterial genera with differential enrichment among the four groups of birds (Fig. 3D). *Romboutsia* and *Turicibacter* were enriched in the CON+ group, while unclassified *Peptostreptococcaceae, Escherichia-Shigella*, and *Chloroplast* were overrepresented in the CON- group. *Lactobacillus* demonstrated higher abundance in the CON-EW group, whereas *Streptococcus* was enriched in the CON-VC group.

A LEfSe comparison of the CON+ group across the two periods revealed five significantly different bacterial genera (Fig. 3E). *Lactobacillus* was more abundant in the CON+ group during P1, while *Turicibacter, Rothia, Candidatus_Arthromitus*, and *Romboutsia* exhibited increased abundance in the same CON+ group during P2.

To further investigate the relative abundance of bacterial genera comparatively in different groups within each period, we used non-parametric tests (Kruskal-Wallis for comparing groups in P1 and P2, and Wilcoxon test for comparing the CON+ group in P1 and P2). The results (i.e. Supplementary Fig. 2-4) were consistent with those obtained from the LEfSe analysis (Fig. 3C-E). As shown in Suppl. Fig. 2, CON+VC reduced relative abundance of *Enterococcus*, while increasing abundance of *Microbacterium, Rothida*, and *Romboutsia* when compared with CON+ in P1. Earthworm supplementation to CON+ diet (i.e. CON+EW) increased relative abundance of *Lactobacillus* and *Microbacterium* as compared with CON+ in P1. In P2, CON- diet reduced relative abundance of *Romboutsia* and *Turicibacter*, while increasing relative abundance of *Escherichia-Shigella, Chloroplast* and unclassified *Peptostreptococcaceae* when compared with CON+ (Suppl. Fig. 3). The supplementation of VC to CON- diet (i.e. CON-VC) increased relative abundance of *Streptococcus* as compared with both CON- and CON+ diets, while decreasing relative abundance of unclassified *Peptostreptococcaceae* in comparison to CON-. The most striking effect of the CON-EW diet was an increased relative abundance of *Lactobacillus* as compared with both CON- and CON+ diets in P2 (Suppl. Fig. 3). The time related changes in the effects of CON+ diet indicated a significant decrease in relative abundance of *Lactobacillus* in P2 as compared to P1, whereas relative abundance of *Turicibacter, Rothia, Candidatus* and *Romboutsia* increased in P2 (Suppl. Fig. 4).

**Fig. 4:**
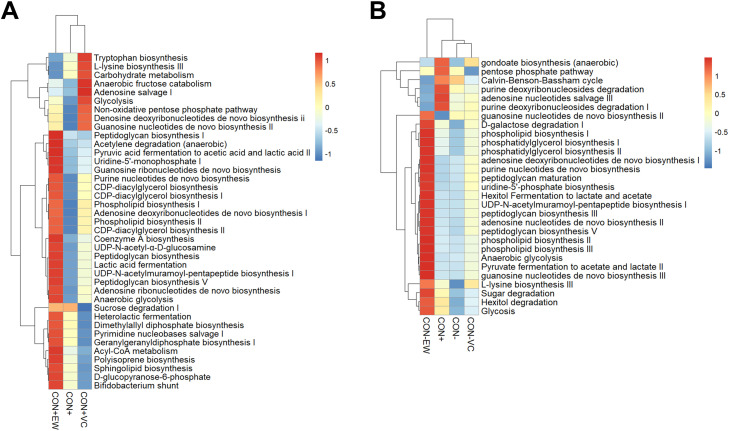
Heatmaps showing two-way hierarchical clustering of predicted metabolic pathways with relative abundances >1 %, as identified by the MetaCyc database. (A) Chickens from Period 1 (CON+, CON+EW, and CON+VC); (B) Chickens from Period 2 (CON+, CON-, CON+EW, and CON+VC). Red indicates higher relative abundances of metabolic pathways, while blue represents lower relative abundances.

### Microbiota functional profiling

The PICRUSt2 analysis, performed using the MetaCyc database, identified a total of 396 metabolic pathways among the microbiota in all experimental treatments during both P1 and P2. The two-way hierarchical clustering of pathways with relative abundances above 1% revealed distinct clustering of groups based on metabolic functions, including carbohydrate metabolism and fermentation, amino acid biosynthesis, lipid metabolism, cofactor and vitamin metabolism, genetic processing, and cell wall component synthesis (Fig. 4). In P1, there were two primary clusters: the CON+ and CON+VC groups were grouped together, with CON+VC exhibiting more enriched pathways related to amino acid biosynthesis and carbohydrate metabolism. In contrast, the CON+EW group formed a separate cluster, characterized by a higher abundance of diverse microbiota metabolic pathways, such as the Bifidobacterium shunt, heterolactic fermentation, sugar degradation I, and glycolysis (Fig. 4A). In P2, four distinct clusters were observed (Fig. 4B). The CON- and CON-VC groups clustered together and displayed relatively lower abundances across all pathways. The CON+EW group formed a separate cluster, showing a unique pattern of increased abundance in most metabolic pathways, particularly those associated with amino acid biosynthesis, carbohydrate degradation and fermentation.

**Fig. 5:**
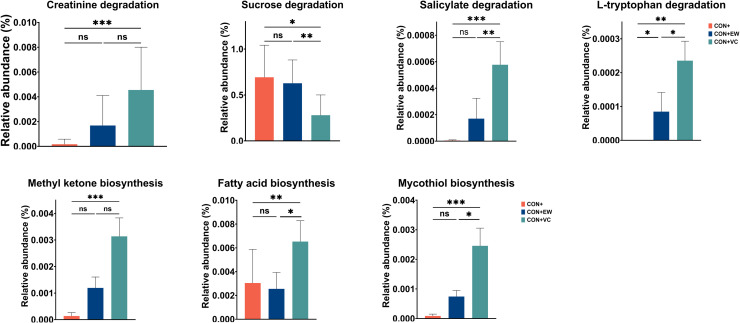
Significantly different pathways in CON+, CON+EW and CON+VC groups during period 1. Differences between groups were assessed using the non-parametric Kruskal-Wallis test, with (*) indicating a significant difference and (ns) denoting non-significance (*P*>0.05).

Kruskal-Wallis test revealed significant differences in seven microbial pathways among groups in P1 (Fig. 5). The CON+VC group exhibited enrichment in microbial pathways associated with the degradation of creatinine, salicylate, and L-tryptophan, along with biosynthesis pathways related to methyl ketone, fatty acids, and mycothiol. The CON+ group showed a higher abundance of the microbial pathway involved in sucrose degradation compared to the CON+VC group, but it did not differ significantly from the CON+EW group.

**Fig. 6:**
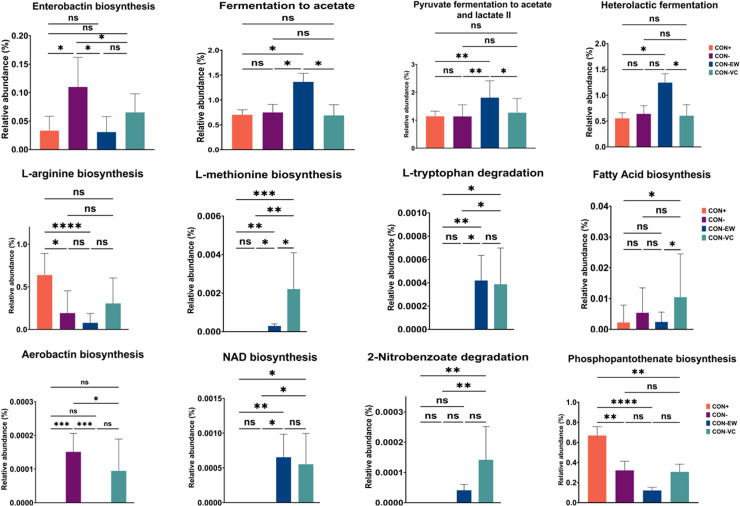
Significantly different pathways among the CON+, CON-, CON-EW, and CON-VC groups during Period 2. Differences between groups were assessed using the non-parametric Kruskal-Wallis test, with (*) indicating a significant difference and (ns) denoting non-significance (*P*>0.05).

In P2, the CON- group demonstrated enrichment in microbial pathways associated with the biosynthesis of enterobactin and aerobactin (Fig. 6). The bacterial communities in the CON-EW group exhibited enrichment in fermentation pathways leading to production of acetate and lactic acid, while the CON-EW group showed a higher relative abundance in microbial pathways related to biosynthesis of L-methionine and fatty acids, as well as degradation pathways for substances such as L-tryptophan and 2-Nitrobenzoate.

### Relationships between differentially abundant bacterial genera and metabolic pathways

Pearson's correlation analysis assessing the relationships between differentially abundant bacterial genera and significantly altered metabolic pathways are summarized in Supplementary Fig. 5. In P1, *Romboutsia* formed a distinct cluster and exhibited positive correlations with several metabolic pathways, including creatinine degradation, L-tryptophan degradation, methyl ketone biosynthesis, and monothiol biosynthesis (Suppl. Fig. 5A). Abundance of *Enterococcus* showed a positive correlation with sucrose degradation, whereas *Lactobacillus* exhibited a negative correlation with this pathway. In contrast, *Microbacterium* demonstrated negative correlations with most metabolic pathways. In P2, *Lactobacillus* formed a separate cluster and positively correlated with metabolic pathways associated with fermentation processes (Suppl. Fig. 5B). Abundance of *Escherichia-Shigella* was positively associated with enterobactin biosynthesis but negatively correlated with pyruvate fermentation to acetate and lactate II. Furthermore, unclassified *Peptostreptococcaceae* exhibited negative associations with fermentation pathways, while *Romboutsia* was positively correlated with L-arginine biosynthesis but negatively associated with fermentation-related pathways.

## Discussion

This study builds on our previous research, which demonstrated the effects of feeding EW and VC on the performance of broiler chickens challenged with an NSP-enriched diet (Daş et al. 2024). The previous study showed that feeding VC enhanced early growth in broilers by increasing feed intake, while feeding EW increased caecal size and reduced the incidence of pasty vent suggesting that EW feeding modulates gut function under challenging dietary conditions. In the present study we substantiated these findings by evaluating microbial composition in the ileum. Our main finding is that the inclusion of VC and particularly EW in broiler diets had notable effects on the ileal microbiota in broilers fed both control and NSP-enriched diets. Firstly, the alpha diversity, particularly species richness (Chao1 index) in P1, was significantly higher in the VC supplemented group suggesting that VC feeding had a rather positive influence on microbial richness, which subsequently transcends into higher feed intake and growth as observed in our previous study (Daş et al. 2024). This could be attributed to the microbial richness in VC (Domínguez et al. 2019), providing a broader range of substrates for microbial fermentation. However, in the second period, when the birds were challenged with NSP-rich diets, species richness was masked even in the VC-fed groups. This observation could indicate that switching to a diet higher in NSP suppresses ileal microbial richness in an elusive manner. The NSP-rich diet seems to have triggered a shift in microbial dynamics, potentially favouring bacteria capable of utilizing NSP over other taxa, thus reducing overall richness. Similarly, our previous study showed that VC supplementation did not promote feed intake and growth when broilers were switched to a diet with a high NSP concentration (Daş et al. 2024). Nevertheless, it is evident that the microbial communities present in the supplemented groups, particularly when fed an NSP-rich diet, are distinct. Notably, the EW- and VC-supplemented groups (CON-EW and CON-VC) displayed diverse microbial profiles compared to the negative control diet (i.e., CON-). The 1 % inclusion level of EW was deliberately chosen to isolate the effects without influencing feed intake. As noted by Bahadori et al. (2017), higher EW levels (above 1%) in the diet may reduce feed intake, potentially confounding the results by altering the microbiome due to decreased consumption, rather than direct effects of EW.

The experimental setup did not include paired control groups in a cross-over design, which would have required incorporating CON+VC and CON+EW treatments in P2 as well. As a control measure to predict time-age-related changes under influences of the same diet, we nevertheless compared CON+ in both P1 and P2. The most pronounced time/age related effect was that *Lactobacillus* became relatively less abundant in P2 than in P1 with the same CON+ diet. This transition implies that microbes other than *Lactobacillus (e.g. Turicibacter, Rothia, Candidatus* and *Romboutsia*) become more abundant as the host ages. Despite the trend towards a reduced abundance of *Lactobacillus* from P1 to P2, EW supplementation of broilers increased relative abundance of *Lactobacillus* with both CON- and CON+ diets in P2, implying a clear microbiota altering effect of EW. The increased relative abundance of *Lactobacillus* in EW fed groups compared to all other groups supports the idea that EW introduces and foster the growth of beneficial microbial populations in the gut (Bahadori et al. 2017), while they can also limit the growth of aerobic pathogenic bacteria as they may constitute peptides with antibacterial activity (Liu et al. 2004). Other authors have indeed reported similar effects when EW meal was fed to broilers. Increased lactic acid producing bacteria and reduced *Escherichia coli* counts in the gut were commonly reported (Bahadori et al. 2017; Loh et al. 2009). *Lactobacillus*—a predominant bacteria genera in host gut—plays a crucial role in gut health by promoting lactic acid production, lowers pH and inhibits the growth of pathogenic bacteria, enhancing gut barrier function and improving overall nutrient absorption (Forte et al. 2016, 2018). This may particularly be helpful in mitigating the challenges induced by dietary NSP. In contrast, birds fed a diet high in NSP without EW supplementation were more enriched with bacterial genera such as *Escherichia-Shigella* which are often associated with pathogenicity. The abundance of these genera may contribute to gut dysbiosis, inflammation and reduced weight gain in broilers (Rubio et al. 2015). The abundance of pathogenic genera in this group might possibly explain or at least be related to the increased incidence of pasty vents as shown in our previous study (Daş et al. 2024). One might also speculate that the higher abundance of *Lactobacillus* induced by EW feeding might have been associated with the reduced incidence of pasty vent in the earthworm fed broilers. Previous research has already made similar association between *Lactobacillus* supplementation and reduced incidence of pasty vent (De Cesare et al. 2017). In addition, the increased caecal size associated with feeding EW to chicks, further suggests that improvement in gut integrity orchestrated by the EW meal reflects enhanced microbial fermentation largely driven by the lactic acid bacteria introduced through the supplementation. However, the exact impact of EW on caecal microbial communities requires further evaluation. Peptostreptococcaceae family was also particularly abundant in the NSP-enriched CON- group but also in those receiving VC (CON-VC). Although not well characterized (Gerritsen et al. 2014), this family is often associated with pathogenicity and has been linked to gut dysbiosis or inflammation (Dehau et al. 2023; Lavelle et al. 2015). Their increase under NSP-induced conditions further suggests that the challenging diet is promoting microbial profiles that might be detrimental to gut health, through unknown mechanisms that needs investigation. This is in line with previous studies highlighting that NSPs in poultry diets can adversely affect nutrient digestibility, compromise immunity and gut health, and increase the susceptibility to intestinal pathogens (Bindari and Gerber, 2022; Immerseel et al., 2004).

The more enriched pathways associated with degradation and biosynthesis particularly in the EW fed group but also mildly in VC fed groups indicate a significant enhancement in microbial metabolic capabilities. These pathway enrichments suggest that feeds supplemented with VC or EW not only promote the growth of beneficial microbial communities but may also improve the resilience of the gut microbiota against potentially harmful conditions. The enrichment of fermentation pathways leading to the production of acetate and lactate further demonstrates the beneficial role of EW feeding. Acetate and lactate are principal end products of intestinal microbial fermentation and serve as important energy sources for intestinal cells and can help lower pH in the gut (Rinttilä and Apajalahti 2013), inhibiting the growth of pathogenic bacteria. In contrast, the NSP enriched groups exhibited increased biosynthesis of enterobactin and aerobactin which are siderophores associated with pathogenic bacteria (Faber and Bäumler 2014). In this study, *Escherichia-Shigella* showed a positive correlation with enterobactin, and the CON- group exhibited a significantly higher abundance of *Escherichia-Shigella*. This shift further suggests the presence of a competitive microbial environment under dietary challenge that could favour the survival of opportunistic pathogens.

The results obtained in this study, though partly speculative, can guide future research. Short-term studies like this, however, do not necessarily reveal subtle changes over time in that the brief duration of the NSP challenge and supplementation may not have captured microbial shift and adaptations. Moreover, there are limits to how far results based on 16S sequencing (taxonomic characterisation of microbes) can go in explaining microbial richness and diversity. PICRUSt2 can predict microbial functions from 16S data and help generate hypotheses where metagenomics is not feasible, they do not replace whole metagenomic sequencing. The methods rely on reference databases, which although continually being updated, may not cover all relevant microbial species and functional pathways rendering it biased. Many predicted functions are based on homologs originally characterized in mammals or humans, which may not fully reflect microbial activity in poultry. Future studies could therefore address these limitations by extending the challenge duration, incorporating higher resolution microbial sequencing, and performing functional validation of microbial activities to ascertain the potential and mechanism by which feeding EW and VC to broilers modulates microbiota and overall gut health particularly under dietary stress.

The use of EW and VC supplements in broiler diets aligns with the principles of circular bioeconomy by recycling organic residues into valuable feed components. Moreover, maintaining a balanced gut microbiota is crucial for overall poultry health, as it supports digestion, immune function, and pathogen resistance. Earthworms enhanced microbial functional diversity and increased abundancy of Firmicutes particularly in the second period, indicating their potential as a sustainable feed ingredient to promote gut health. The observed modulation of ileal microbiota under NSP-enriched diets suggests that EW may provide resilience against dietary challenges, potentially improving bird performance and feed efficiency in the longer term. In conclusion, this study demonstrates the potential of dietary EW supplementation to positively influence gut microbiota composition and function, particularly in response to dietary challenges.

## Data availability

The 16 S rRNA gene sequencing data used in this study are available at ht tps://zenodo.org/records/14226257

## CRediT authorship contribution statement

**Muhammad Zeeshan Akram:** Writing – original draft, Data curation, Formal analysis, Investigation, Methodology, Software, Visualization. **Oyekunle John Oladosu:** Writing – original draft, Investigation, Methodology. **Nadia Everaert:** Conceptualization, Funding acquisition, Methodology, Resources, Supervision, Writing – review & editing. **Cornelia C. Metges:** Conceptualization, Methodology, Funding acquisition, Resources, Supervision, Writing – review & editing. **Gürbüz Daş:** .

## Declaration of competing interest

The authors declare that they have no competing interests.

## Appendix Supplementary materials

Image, application 1
