# Supplementary material for: Modulation of early life gut microbiota through inclusion of earthworms and vermicompost in broiler diets
Source: Poult Sci. 2025 Apr 1;104(6):105121. doi: 10.1016/j.psj.2025.105121 (PMC12001109; doi:10.1016/j.psj.2025.105121)
Supplement: Supplementary file 1 [file mmc1.docx]

**SUPPLEMENTARY MATERIALS**

**Supplementary Table 1**. Ingredients and analyzed chemical composition of the experimental diets and earthworms (EW).

| **Item** | **CON+** | | **CON-** | **CON+VC** | **CON-VC** | **EW** |
| --- | --- | --- | --- | --- | --- | --- |
| **Ingredients, g/kg as fed** | | | | | | |
| Corn | | 580.85 | 278.85 | 533.80 | 229.70 | - |
| Soybean meal (480 CP) | | 349.00 | 334.00 | 354.00 | 340.00 | - |
| Soybean oil (8957 kcal) | | 25.00 | 41.00 | 40.00 | 57.00 | - |
| Wheat | | 0.00 | 100.00 | 0.00 | 100.00 | - |
| Barley | | 0.00 | 100.00 | 0.00 | 100.00 | - |
| Rye | | 0.00 | 100.00 | 0.00 | 100.00 | - |
| Vermicompost**^1^** | | 0.00 | 0.00 | 28.25 | 28.25 | - |
| Premix**^2^** | | 5.00 | 5.00 | 5.00 | 5.00 | - |
| Lime fine | | 14.80 | 14.80 | 13.40 | 13.20 | - |
| Monocalcium phosphate | | 14.40 | 14.00 | 14.40 | 14.40 | - |
| NaCl | | 1.90 | 1.90 | 1.90 | 1.90 | - |
| NaHCO_3_ | | 2.80 | 2.80 | 2.80 | 2.80 | - |
| L-Lysine HCl | | 2.00 | 2.45 | 1.95 | 2.40 | - |
| DL-Methionine | | 3.15 | 3.40 | 3.25 | 3.45 | - |
| L-Threonine | | 0.80 | 1.15 | 0.85 | 1.15 | - |
| L-Valine | | 0.30 | 0.65 | 0.40 | 0.75 | - |
| **Chemical analysis, g/kg DM** | | | | | | |
| Dry matter, g/kg | | 890 | 894 | 882 | 887 | 145 |
| Crude protein | | 242 | 243 | 247 | 251 | 630 |
| Crude fat | | 57 | 67 | 72 | 80 | 62 |
| Starch**^3^** | | 457 | 418 | 430 | 385 | 22 |
| Sugars | | 38 | 43 | 41 | 43 | 18 |
| Crude fiber | | 22 | 27 | 32 | 39 | 22 |
| NDF | | 126 | 164 | 130 | 167 | 63 |
| ADF | | 53 | 52 | 49 | 54 | 52 |
| Crude ash | | 67.5 | 66.0 | 68.5 | 69.5 | 83 |
| Soluble NSP | | 19.0 | 25.5 | 21.4 | 27.4 | - |
| Insoluble NSP | | 92.7 | 99.4 | 83.2 | 99.0 | - |
| Total NSP**^4^** | | 111.7 | 124.9 | 104.6 | 126.4 | - |
| Ca | | 11.8 | 10.0 | 10.4 | 10.5 | 7.1 |
| P | | 7.6 | 7.2 | 7.2 | 8.0 | 9.4 |
| Mg | | 2.0 | 1.9 | 2.1 | 2.1 | 2.1 |
| K | | 7.6 | 10.9 | 11.1 | 10.9 | 11.3 |
| Na | | 1.7 | 1.8 | 2.0 | 1.9 | 6.1 |
| ME, kcal/kg DM | | 3,298 | 3,251 | 3,346 | 3,251 | 2,980 |

Each value represents average of two analyzed samples.

**^1^** DM of vermicompost (VC) was 35.4%. In order to ensure 1% VC in the DM of the supplemented diets, 28.25 g VC (as is) was included in 1000 g of the supplemented diets.

**^2^** Ingredients per kg premix: (see Daş et al., 2024).

**^3^** For earthworms it is glycogen.

**^4^** For details of individual sugars and sugar acids as components of NSP see Daş et al., 2024.

**Abbreviations**: **CON+**: positive control diet; **CON-**: negative control diet; **CON+VC**: positive control diet supplemented with 1% vermicompost; **CON-VC**: negative control diet supplemented with 1% vermicompost in dry matter; **EW**: Earthworm.


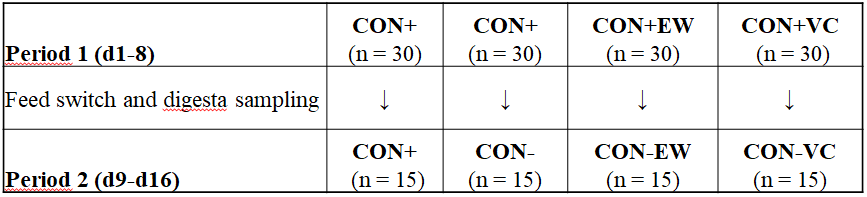


**Supplementary Figure 1:** Experimental design with the diets fed to broilers in two feeding periods separated by a feed switch.

Total number of birds sampled for ileal digesta sampling in this study was N = 120 (i.e. 15 birds / group in each period).

Number of samples used for statistical analyses after DNA quality control was as following: Period 1: CON+ (n=9), CON+EW (n=12), CON+VC (n=13); Period 2: CON+ (n=10), CON- (n=9), CON-EW (n=13), and CON-VC (n=10).

**Abbreviations**: **CON+**: positive control diet; **CON-**: negative control diet; **CON+EW**: positive control diet plus 1% of earthworm; **CON+VC**: positive control diet supplemented with 1% vermicompost; **CON-EW**: negative control diet plus 1% earthworm; **CON-VC**: negative control diet supplemented with 1% vermicompost.

**Supplementary Figure 1:** Experimental design with the diets fed to broilers in two feeding periods separated by a feed switch. Total number of birds used in this study (N = 120).


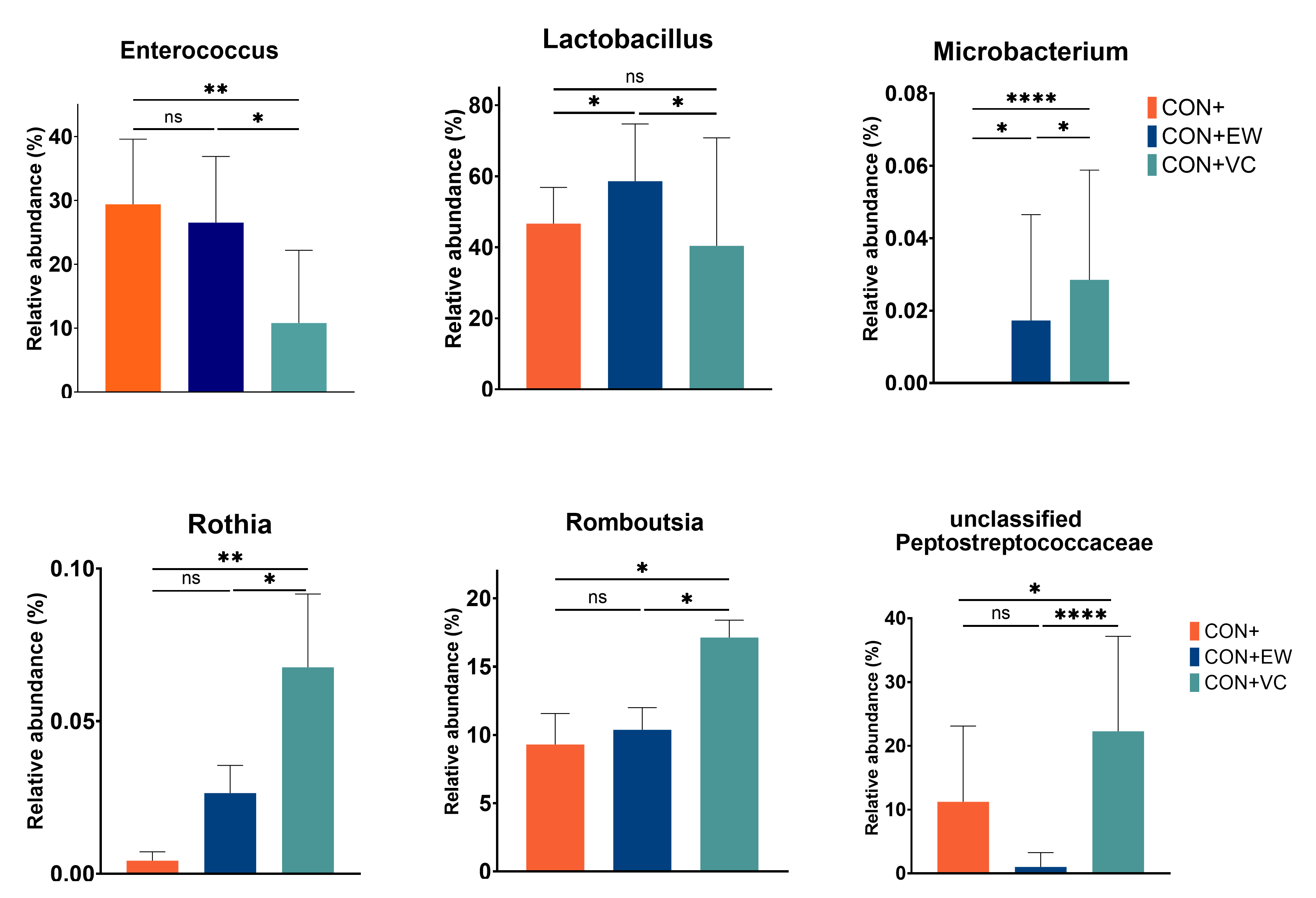


**Supplementary Figure 2:** Relative abundance of differentially abundant bacterial genera in the CON+ (n = 9), CON+EW (n = 12), and CON+VC (n = 13) groups during Period 1. Statistical significance between groups was assessed using the non-parametric Kruskal-Wallis test, with (*) indicating a significant difference and (ns) denoting non-significance (*P*>0.05).


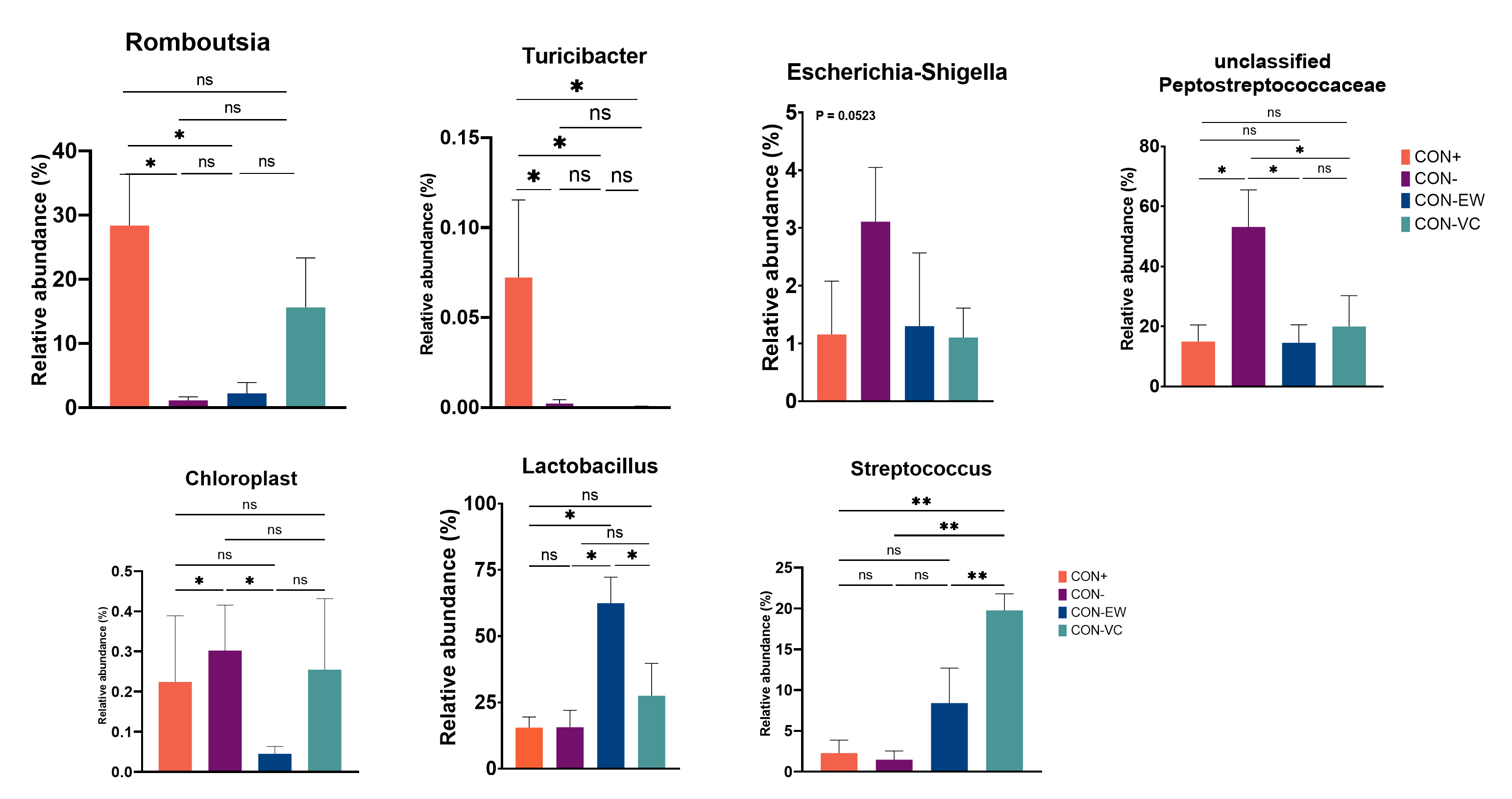


**Supplementary Figure 3:** Relative abundance of differentially abundant bacterial genera in the CON+ (n = 10), CON- (n = 9), CON-EW (n = 13), and CON-VC (n = 10) groups during Period 2. Statistical significance between groups was assessed using the non-parametric Kruskal-Wallis test, with (*) indicating a significant difference and (ns) denoting non-significance (P>0.05).


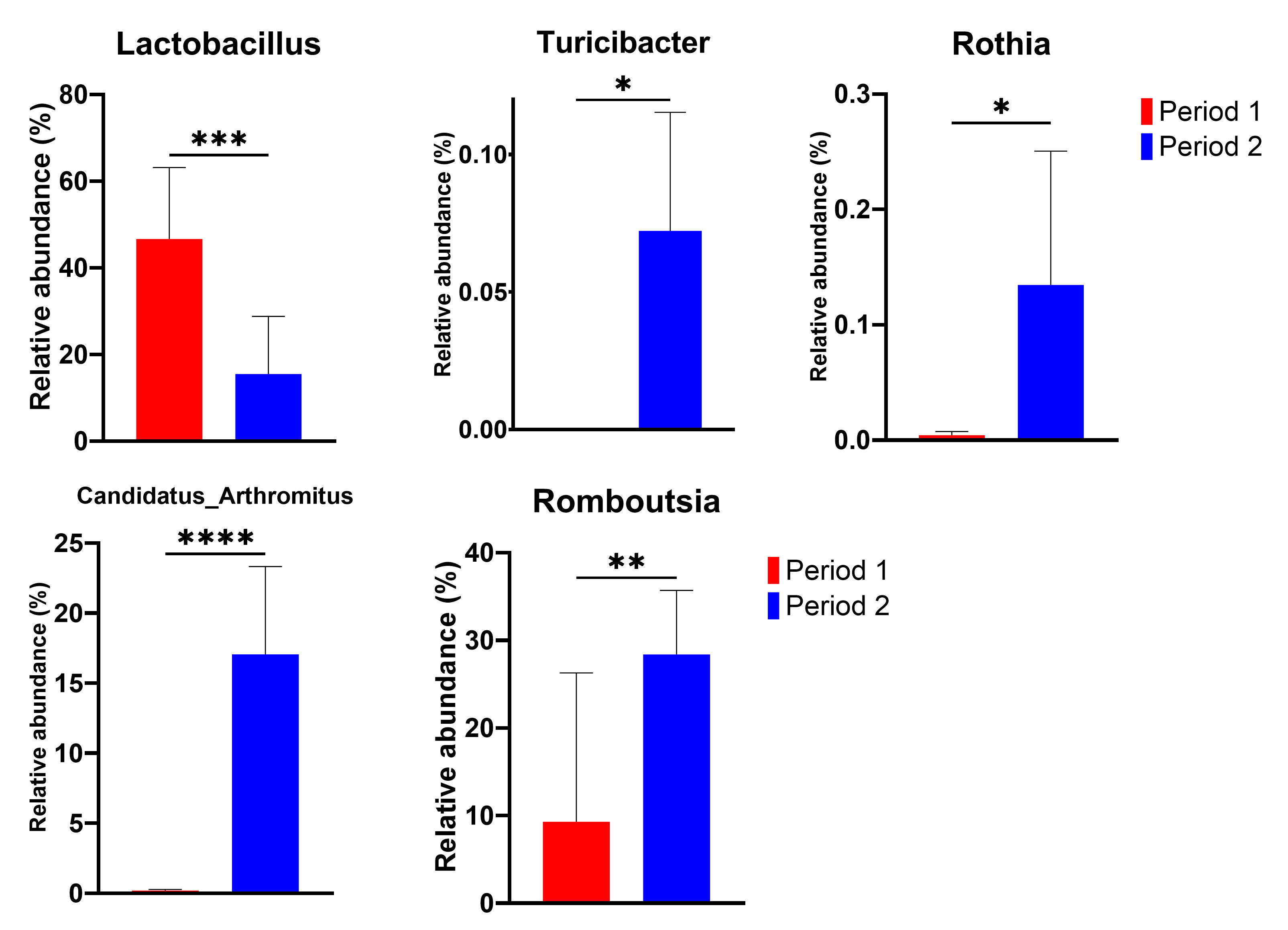


**Supplementary Figure 4:** Relative abundance of differential bacterial genera in the CON+ group across two periods (P1: n = 9 and P2: n = 10). Statistical significance between groups was assessed using the non-parametric Wilcoxon test, with (*) indicating a significant difference (P<0.05).


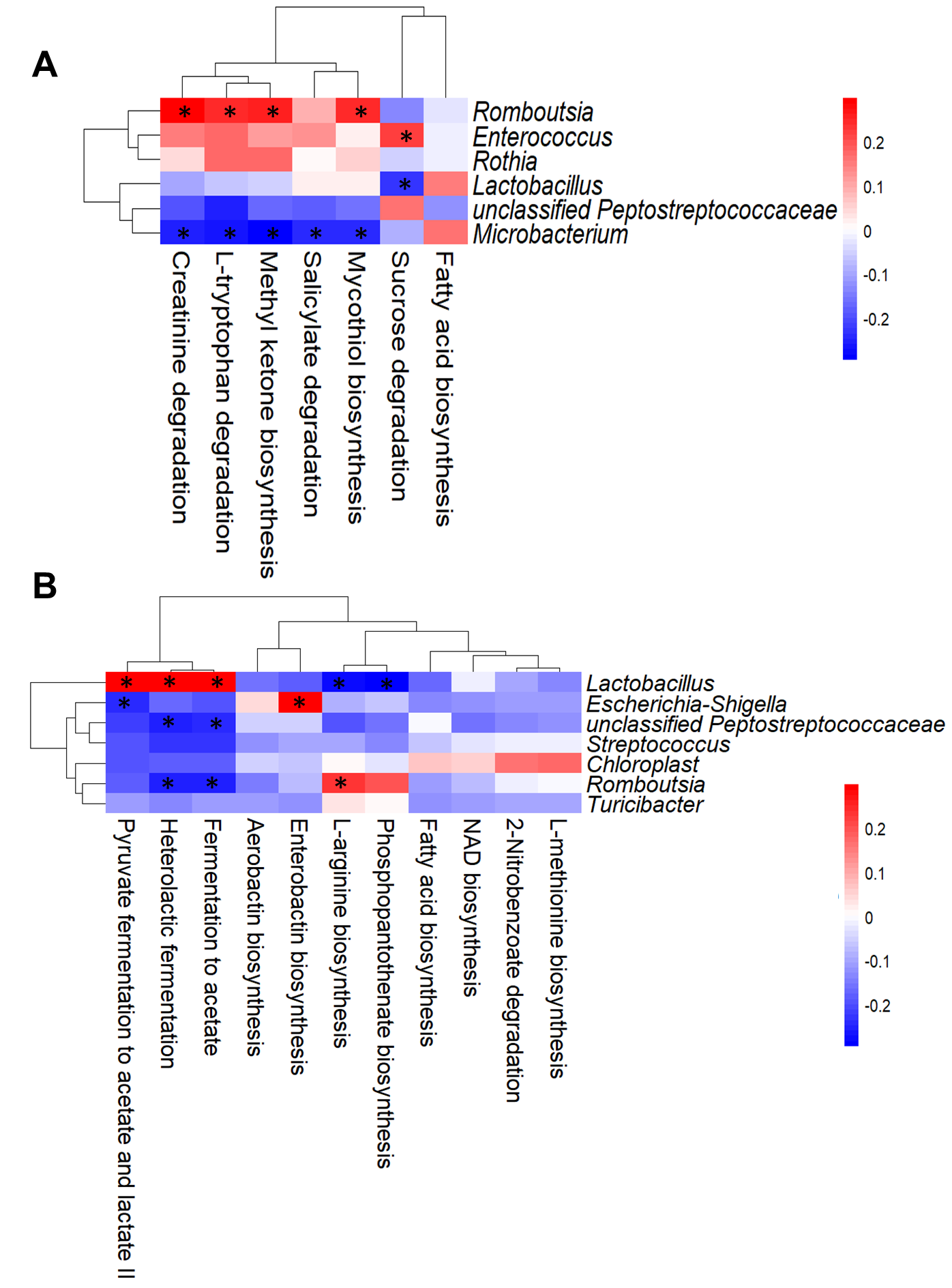


**Supplementary Figure 5:** Heatmaps presenting relationships between differentially abundant bacterial genera and significantly altered predicted metabolic pathways, using data pooled across three groups in P1 and four groups in P2. Significant correlations (P < 0.05) are marked with an asterisk, with red representing positive correlations and blue representing negative correlations.
